# Interaction of dynamic error signals in saccade adaptation

**DOI:** 10.1152/jn.00419.2022

**Published:** 2023-02-15

**Authors:** Ilja Wagner, Alexander C. Schütz

**Affiliations:** ^1^AG Allgemeine und Biologische Psychologie, Philipps-Universität Marburg, Marburg, Germany; ^2^Center for Mind, Brain and Behavior, Marburg, Germany

**Keywords:** implicit and explicit learning, motor adaptation, saccades

## Abstract

Motor adaptation maintains movement accuracy. To evaluate movement accuracy, motor adaptation relies on an error signal, generated by the movement target, while suppressing error signals from irrelevant objects in the vicinity. Previous work used static testing environments, where all information required to evaluate movement accuracy was available simultaneously. Using saccadic eye movements as a model for motor adaptation, we tested how movement accuracy is maintained in dynamic environments, where the availability of conflicting error signals varied over time. Participants made a vertical saccade toward a target (either a small square or a large ring). Upon saccade detection, two candidate stimuli were shown left and right of the target, and participants were instructed to discriminate a feature on one of the candidates. Critically, candidate stimuli were presented sequentially, and saccade adaptation, thus, had to resolve a conflict between a task-relevant and a task-irrelevant error signal that were separated in space and time. We found that the saccade target influenced several aspects of oculomotor learning. In presence of a small target, saccade adaptation evaluated movement accuracy based on the first available error signal after the saccade, irrespective of its task relevance. However, a large target not only allowed for greater flexibility when evaluating movement accuracy, but it also promoted a stronger contribution of strategic behavior when compensating inaccurate saccades. Our results demonstrate how motor adaptation maintains movement accuracy in dynamic environments, and how properties of the visual environment modulate the relative contribution of different learning processes.

**NEW & NOTEWORTHY** Motor adaptation is typically studied in static environments, where all information that is required to evaluate movement accuracy is available simultaneously. Here, using saccadic eye movements as a model, we studied motor adaptation in a dynamic environment, where the availability of conflicting information about movement accuracy varied over time. We demonstrate that properties of the visual environment determine how dynamic movement errors are corrected.

## Graphical abstract

**Figure:**
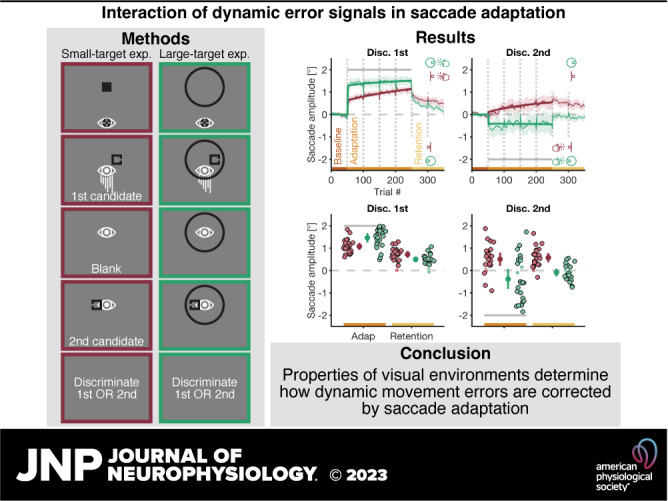

## INTRODUCTION

Humans use a repertoire of different motor actions to interact with the world around them. To allow for goal-directed interactions with the world over a life time, the brain constantly monitors the spatial accuracy of every motor action, and, if necessary, recalibrates the corresponding motor command to compensate for perturbations (e.g., pathologies, aging, or muscle fatigue) to movement accuracy (e.g., see Refs. 1–3)—a process referred to as motor adaptation (for a review, see Ref. 4).

To study motor adaptation in the laboratory, movement accuracy of different effectors can be perturbed artificially. For this, force-fields can be applied to ongoing reaching movements [sensorimotor adaptation (e.g., see Ref. 5)], the mapping between online visual feedback and an ongoing motor action can be altered [visuomotor adaptation (e.g., see Ref. 6)], or the spatial position of an eye movement target can be manipulated while a saccadic eye movement is in flight [saccade adaptation (e.g., see Ref. 7, for reviews see Refs. 8 and 9)]. In all those examples, an error signal is generated either during the ongoing movement, in case of manual actions, or after the movement, in case of eye movements, which is then processed by the cerebellum, and used to evaluate movement accuracy (for reviews, see Refs. 8, 10, and 11). For saccadic eye movements specifically, a recent study demonstrated that motor adaptation is based on a comparison between the expected and actual sensory consequences of saccades, from which a motor error is estimated after saccade termination, and that this postdicted error drives motor learning (12), instead of a pure prediction error, which was previously assumed to be the main driving force behind saccade adaptation (13, 14). However, retinal errors (i.e., the distance between the fovea and the position of the eye movements target after a saccade) have also been found to be effective in driving adaptation of saccadic eye movements and are often used as to study how the brain corrects spatially inaccurate saccades (for a review, see Ref. 8).

Motor adaptation in general is typically studied in relatively simple laboratory settings, where a single, isolated error signal provides unambiguous feedback about movement accuracy. However, some recent studies began to use more complex environments, where the brain has to resolve conflicts between multiple competing error signals (15–20), and dynamic environments, where motion of a movement target generates an error signal about movement accuracy (21). For saccade adaptation, Madelain et al. (20), for example, asked participants to make a saccade toward an eye movement target, which was placed on an image of a naturalistic scene. The authors demonstrated that saccade adaptation filters out any error signal that was generated by a perisaccadic position change of the background, while selectively utilizing the error signal from the eye movement target to evaluate movement accuracy. Saccade adaptation only utilized error signals from the background image when the eye movement target was turned off upon saccade onset, i.e., in the absence of any clearly defined saccade target. Similar effects can be observed when a distractor stimulus is shown together with the eye movement target after a saccade (17). Here, saccade adaptation selectively filters out the error signal from the distractor stimulus (17), unless the distractor stimulus is rendered highly salient (16).

Several studies also highlighted how the requirements of a behavioral or perceptual task can influence which error signal is used in saccade adaptation to evaluate movement accuracy in complex visual environments (22–25, for a review, see Ref. 26). For example, instructing participants to discriminate one stimulus within an array of several task-irrelevant distractors (23, 24) generates a task-related error signal that is used by saccade adaptation to ensure that task-relevant visual information in the array is foveated. Similar effects can be observed when participants have to report the orientation of two simultaneously shown stimuli sequentially (25) or when participants are merely instructed about what part of a stimulus array to track with their gaze (22). Thus, in all those cases, requirements of the respective task determine which error signal is used in saccade adaptation to evaluate movement accuracy, and which error signals are filtered out.

Using saccades as a model for motor adaptation, a variety of studies, thus, demonstrated how motor adaptation operates in stimulus-rich environments, where conflicting feedback about movement accuracy generates competing error signals. However, studies that tested how motor adaptation resolves conflicts between multiple competing error signals thus far mostly used static testing environments, where all information that is required to evaluate movement accuracy was either available during the ongoing movement (reaching movements) or shortly after movement termination (saccades). Natural environments, however, are often dynamic, and visual information, relevant to estimate movement accuracy, can become temporarily obscured, or drift in and out of view over time. In complex situations like this, the motor system must selectively process the right error signal (i.e., the one most relevant for the current behavioral goal) at the right time (i.e., when it becomes available) to maintain movement accuracy in an ever-changing world.

Previous studies reported evidence that delaying postsaccadic feedback about eye movement accuracy leads to gradually weaker saccade adaptation with increasing delay duration, and that saccade adaptation vanishes when the temporal delay between saccade offset and feedback about movement accuracy is too long (27–29). However, those studies used task-irrelevant eye movement targets, and participants were merely required to make a saccade toward a stimulus, without the need to interact with or to extract visual information from the saccade target. Assigning task-relevance to saccade targets allows to extend the critical time window, in which saccade adaptation evaluates movement accuracy, such that adaptation occurs even when error signals become available long after saccade offset (30).

We investigated how the brain resolves conflicts between temporarily dynamic error signals in two different visual environments, using saccadic eye movements as a model for motor adaptation. More specifically, using visual environments with either a large or a small saccade target, we tested if an error signal, generated by the difference in the saccade landing position and the location of a task-irrelevant stimulus immediately after the eye movement, can be suppressed for the benefit of a temporally delayed error signal, generated by the difference in saccade landing position and the location of a task-relevant stimulus.

## METHODS

### Participants

We recorded data from 29 participants in the large-target experiment and data from 26 participants in the small-target experiment. Four participants in the large-target experiment and two participants in the small-target experiment had to be excluded because they voluntarily opted out before finishing all sessions. In addition, one participant in the large-target experiment had to be excluded because more than 50% of his/her trials had to be excluded from data analysis (see “*Data exclusion*” for exclusion criteria) across multiple sessions, despite extensive training with the task and re-measurement of sessions with an excessively large proportion of excluded trials. Due to this, data collection for this participant was not continued beyond his/her second session. The remaining 24 participants in the large-target experiment had a mean age of 24 yr (min = 19 yr, max = 40 yr, 18 females). In the small-target experiment, the remaining 24 participants had a mean age of 23.46 yr (min = 18 yr, max = 31 yr, 13 females).

In the large-target experiment, one participant had to repeat two sessions due to low data quality, and one additional participant had to repeat one session for the same reason. In the small-target experiment, one participant had to repeat two sessions due to low data quality, three participants had to repeat one session for the same reason, and one additional participant had to repeat one session due to technical difficulties. Whenever sessions were repeated, we ensured that at least between five and seven days passed between the original and the repeated session.

All participants provided written informed consent before testing, were naïve as to the purpose of the experiments, and had normal or corrected-to-normal vision. Experiments were conducted in accordance with the ethical guidelines laid down in the 1964 declaration of Helsinki and were approved by the ethics committee of the Marburg University, Department of Psychology (Proposal 2017-27k). Participants in the large-target experiment were compensated with 8 €/h; participants in the small-target experiment could choose if they wanted to be compensated with course credits or monetarily. After successful completion of all four sessions, participants in the large-target experiment received 8 € as an additional bonus payment. Participants in the small-target experiment received an additional bonus payment of either one course credit or 8 €, depending on what they chose as their preferred means of compensation.

### Equipment

Both experiments were conducted using the Psychtoolbox (31–33) in MATLAB R2016a (The MathWorks, Natick, MA). Stimuli were presented on a VIEWPixx monitor (VPixx Technologies Inc., Saint-Bruno, QC, Canada), while participants maintained a viewing distance of 60 cm. The monitor had a size of 51.50 × 29 cm, a spatial resolution of 1,920 × 1,080 pixels and a refresh rate of 120 Hz. The background color was set to gray (R: 128, G: 128, B: 128; luminance 54.6 cd/m^2^). Eye movements of the right eye were recorded with an EyeLink 1000+ (SR Research Ltd., ON, Canada) at a sampling rate of 1,000 Hz. Data of two sessions was accidentally recorded with a sampling rate of 2,000 Hz, and down sampled to 1,000 Hz before offline analysis. The Eyelink Toolbox was used for control of the eye tracker (34).

### Stimuli

A combination of cross and bull’s eye (total diameter: 0.60°) was used as fixation target (35). A large ring [outline width: 0.15°, ring diameter: 6.50°, outline color: (R: 0, G: 0, B: 0); luminance 0.39 cd/m^2^] was used as eye movement target in the large-target experiment, whereas a smaller, filled square was used in the small-target experiment [square width: 1°, fill color: (R: 0, G: 0, B: 0)]. In both experiments, two unfilled squares [outline width: 0.15°, square width: 1°, outline color: (R: 0, G: 0, B: 0)] were used as candidate stimuli. Each candidate stimulus had a gap [gap width: 0.03°, fill color: (R: 38, G: 38, B: 38); luminance 15.40 cd/m^2^] at one of its four sides.

The fixation cross was shown centered on the horizontal screen axis, while being slightly offset relative to the vertical screen axis (2° beneath vertical screen center). The eye movement target was presented centered on the horizontal screen axis, and at a vertical eccentricity of 10° relative to the location of the fixation cross. Candidate stimuli appeared at a horizontal eccentricity of either +2° or –2° relative to screen center, and at the same vertical eccentricity as the eye movement target.

### Design

We used a mixed design with two experiments (large-target and small-target experiment) and four conditions (discriminate-1st, discriminate-2nd, discriminate-1st-no-2nd, discriminate-2nd-no-1st condition). Experiments were treated as a between-subject factor, whereas conditions were treated as a within-subject factor. Each condition had 350 automatically paced trials and was divided into three phases: a baseline phase (50 trials), an adaptation phase (200 trials), and a retention phase (100 trials). In both experiments, data for the respective four conditions was collected in four separate sessions, with each session being recorded on separate days. It was ensured that at least five to seven days passed between each session [large-target experiment: mean (*M*) = 8.56 days, min = 5.33 days, max = 32.67 days; small target: *M* = 7.32 days, min = 5 days, max = 23.67 days).

Each participant completed the four conditions in an individual order, which was determined by, first, calculating all possible permutations in which the four conditions could be completed (i.e., 4!), and second, assigning each participant to one of the resulting unique permutations. We balanced if the first candidate stimulus appeared at a horizontal eccentricity of +2° or –2° across participants, so that it appeared left of the horizontal screen center for half of the participants, and right of the horizontal screen center for the other half. Within each participant, the horizontal locations of the first and second candidate stimulus were kept constant across conditions.

Participants in all conditions of both experiments completed 15 demonstration trials (five baseline and ten adaptation trials) before the start of each condition.

### Procedure

#### Discriminate-1st condition.

**Figure 1.**
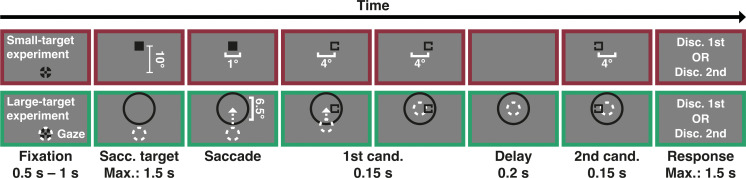
Trial procedure in adaptation trials of the small-target (*top row*) and large-target experiment (*bottom row*). Participants were instructed to make a vertical saccade to an eye movement target (either a small square or a large ring). Upon saccade detection, a candidate stimulus was shown at an eccentric location, followed by a temporal delay, and a second candidate stimulus that was shown at the opposite location of the first candidate stimulus. After offset of the last candidate stimulus, participants were instructed to discriminate the gap location on either the first (discriminate-1st condition) or the second (discriminate-2nd condition) candidate stimulus. The eye movement target in the large-target experiment was shown until onset of the response phase, whereas it was turned off upon saccade detection in the small-target experiment. Stimuli are not drawn to scale. White elements are for illustration only and were not visible to participants.

In all trials of the discriminate-1st condition, a fixation target appeared at trial start, which participants were instructed to look at (Fig. 1). After a random time-interval between 500 ms and 1,000 ms, drawn from a uniform distribution, an eye movement target was shown, and the fixation target was turned off. Participants were instructed to make an upward saccade toward the eye movement target after its onset.

In adaptation trials, the first candidate stimulus was shown close to the eye movement target’s location upon saccade detection. The first candidate stimulus stayed on for 150 ms. After a temporal delay of 200 ms, a second candidate stimulus appeared at the opposite location of the first candidate stimulus (i.e., if the first candidate stimulus was shown at a horizontal eccentricity of +2°, the second candidate stimulus was shown at −2°) and stayed on for 150 ms. Since the first candidate stimulus was presented upon online detection of primary saccade onset, and thus while the saccade was still in flight, the net viewing time of the first candidate stimulus was slightly shorter (by ∼30 ms) compared with the net viewing time of the second candidate stimulus. The duration of the temporal delay between offset of the first and onset of the second candidate stimulus was chosen for three reasons. First, we wanted to test error evaluation for saccade adaptation at the end of the sensitive time window. Second, we wanted to ensure that participants have sufficient time to program a corrective saccade toward the second candidate stimulus if this stimulus was not chosen as a target for the primary saccade. Third, we wanted to ensure that participants perceive the two candidate stimuli as two separate entities that appear at different points in time, instead of being parts of a singular event. After the offset of the second candidate stimulus, all stimuli were removed from the screen, and participants had to report which side of the first candidate stimulus (top, bottom, left, right) had a gap. For this, they used the arrow keys on the keyboard and were given up to 1,500 ms for their response.

After a response was placed or if a response timeout occurred, either the next trial began automatically, or one of two feedback sounds was presented for 500 ms: a low-pitched sound (400 Hz) was played if participant’s perceptual judgment was incorrect or if they did not give any response within the provided timeframe. A high-pitched sound (1,500 Hz) was played if participants broke fixation prematurely or if they made no saccade within 1,500 ms after onset of the eye movement target (saccade timeout). When saccade timeouts occurred, all stimuli were removed from the screen, no candidate stimuli were presented, and participants were not required to place a perceptual judgment at the end of a trial. Otherwise, the trial progressed as if participants made a saccade.

Baseline and retention trials were identical to adaptation trials, with the only differences being that no candidate stimuli were shown after the primary saccade, and participants thus were not required to place perceptual judgments. We decided to not show any candidate stimuli in retention trials, because we were interested in measuring how much of the behavior that participants learned in adaptation trials will be retained in the absence of any error signal. By presenting a postsaccadic stimulus (e.g., showing a candidate stimulus at a central location) we would no longer measure retention, but instead adaptation toward this central stimulus. The timing in baseline and retention trials was matched to the timing in adaptation trials.

After each 50th trial, a 30-s set break was presented, during which the screen turned black, and participants were instructed to close their eyes. Twenty-five seconds (25 s) into a set break, a sound with an intermediate pitch (700 Hz) was presented, notifying participants that the set break is about to end, and that they can open their eyes again. From this point, the screen turned gray again, a fixation cross was presented, and, after five more seconds, the next trial began automatically. A similar procedure was previously used in other studies on saccade adaptation (e.g., see Refs. 23–25, 36, and 37).

#### Discriminate-2nd condition.

The discriminate-2nd condition was identical to the discriminate-1st condition, with the only difference being that participants were instructed to report the gap position on the second candidate stimulus.

#### Discriminate-1st-no-2nd and discriminate-2nd-no-1st conditions.

The discriminate-1st-no-2nd and discriminate-2nd-no-1st condition were identical to the discriminate-1st and discriminate-2nd condition, respectively, with the only difference being that only one (either the first or the second) candidate stimulus was shown. The timing in both conditions was matched to the timing in the discriminate-1st and the discriminate-2nd condition, i.e., the candidate stimulus in the discriminate-1st-no-2nd condition was shown upon saccade detection, whereas it appeared 350 ms after online saccade detection in the discriminate-2nd-no-1st condition.

#### Differences between small-target and large-target experiment.

The trial procedure in the small-target (Fig. 1, *top row*) and large-target experiment (Fig. 1, *bottom row*) was mostly identical, except for two differences: First, a large ring was used as eye movement target in the large-target experiment, whereas a small, filled square was used in the small-target experiment. Consequently, the candidate stimuli appeared within the eye movement target in the large-target experiment, whereas candidate stimuli appeared left and right of the eye movement target’s position in the small-target experiment. Second, the eye movement target in the large-target experiment stayed on until onset of the response phase, whereas it was turned off upon online saccade detection in the small-target experiment. Since the large-target experiment was intended to test how a large attentional locus influences target selection for saccade adaptation, the eye movement target in this experiment stayed on until the onset of the response phase to ensure that participants maintain their attentional locus until the last candidate stimulus was presented.

### Eye Movement and Data Analysis

#### Detection of primary and secondary saccades.

Saccade on- and offsets were detected using the EyeLink algorithm. Primary saccades were defined as the first saccade after eye movement target onset that brought gaze outside a circular area (radius: 2°) around the fixation cross. Secondary saccades were defined as the first saccade in a trial that was made between offset of the primary saccade, and up until 60 ms after the onset of the second candidate stimulus. Secondary saccades were excluded from analysis if their vertical end point was below 5° or if their absolute horizontal end point was above 6°, i.e., if the secondary saccade likely brought gaze back to screen center or targeted something other than a candidate stimulus.

#### Analysis of saccade acceleration and deceleration.

The analysis of horizontal displacement during the primary saccade’s acceleration and deceleration phase was based on the procedure reported by Orozco et al. (38). First, we calculated the two-dimensional (2-D) velocity vector of primary saccades (vel_ps_) as ${\text{vel}}_{\text{ps}}=\sqrt{{\text{vel}}_{\text{h}}^{2}+{\text{vel}}_{\text{v}}^{2}}$. Here, vel_h_ and vel_v_ are the horizontal and vertical velocity of the primary saccade, respectively. Taking the maximum of vel_ps_ yields the primary saccade’s peak velocity, which we then used to divide the horizontal saccade trace into an acceleration phase (i.e., the trace segment before peak velocity) and a deceleration phase (i.e., the trace segment after peak velocity). Calculating the horizontal saccade amplitude component in the acceleration and deceleration phase yields the horizontal displacement during those two phases.

#### Baseline correction.

Baseline correction was applied to horizontal primary saccade amplitudes, and horizontal displacement during a saccade’s acceleration and deceleration phase. For this, we, calculated the mean value of each variable in baseline trials, and subtracted the resulting mean from the corresponding variable’s value in each trial. All analyses in the manuscript were performed on baseline corrected data.

#### Moving average.

For illustration purposes, moving averages of horizontal primary saccade amplitudes as well as horizontal displacement during saccade acceleration and deceleration were calculated for each participant and each block of trials (one block of trials corresponds to 50 trials) individually, by sliding a moving window with variable bin width over the data. For the first bin, the moving window had a width of two trials, whereas its width was four trials for the second bin, and six trials for all subsequent bins. Only trials right to the current sample were considered when calculating the average of a bin, hence, an asymmetrical moving window was used. This procedure was used to accentuate the rapid changes in saccade amplitude that occur at onset of set breaks, and was based on the procedure reported by Ethier et al. (37). For consistency, the same procedure was applied to all instances where a moving average is shown.

#### Statistical analysis.

**Figure 2.**
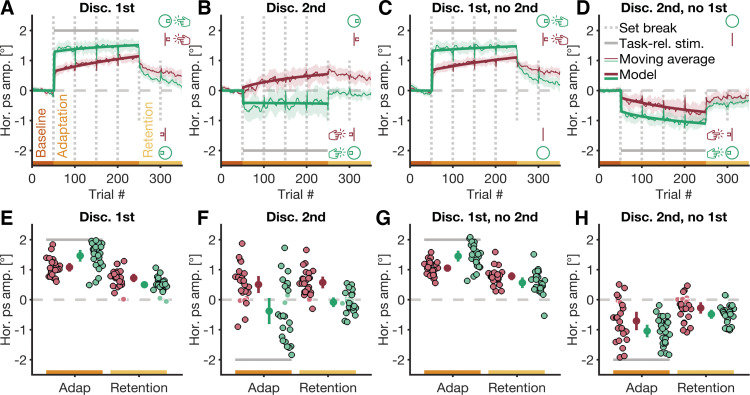
Adaptation time courses and mean horizontal saccade amplitudes. *A*–*D*: horizontal amplitudes of primary saccades (Hor. ps amp.) in the four conditions (Discriminate-1st, Discriminate-2nd, Discriminate-1st-no-2nd, Discriminate-2nd-no-1st) of the small-target (red) and large-target experiment (green). Thin colored lines are moving averages over average horizontal amplitudes, and thick colored lines represent the horizontal saccade amplitude, as predicted by our computational model. The model was only fitted to baseline and adaptation trials, but not to retention trials. Dotted gray lines are set breaks. The shaded areas around data points are 95% confidence intervals of moving averages. *E*–*H*: mean horizontal saccade amplitudes in the last block of adaptation trials (Adap)/the first block of retention trials (Retention). Large datapoints are averages across participants, small datapoints are data of individual participants. Black and white outlines around data points of individual participants mark participants for whom the average horizontal amplitude in the corresponding block of trials was or was not significantly different from zero (see *Eye Movements and Data Analysis* for the procedure). Error bars are 95% confidence intervals. *A*–*H*: thick gray lines mark the location of the task-relevant candidate stimulus in adaptation trials (Task-rel. stim.). Positive values on the *y*-axis correspond to saccade amplitudes in direction of the first candidate stimulus, whereas negative values correspond to amplitudes in direction of the second candidate stimulus.

Since all our dependent variables violated at least one assumption for an ANOVA, we used robust two-way mixed ANOVAs with 20% trimmed means for statistical inference. The robust ANOVA was part of the WRS2 package (39) for R (R Core Team, 2019). The normality assumption was tested with the Shapiro–Wilk test, sphericity was tested with Mauchly’s *W* test, and variance homogeneity was tested using Levene’s test. Outliers were detected by inspecting QQ-plots and Boxplots. The significance of the adaptation magnitude of individual participants was tested by calculating, for each participant individually, the 95% confidence interval around the individual mean horizontal primary saccade amplitude. If the resulting confidence interval included zero, the null hypothesis was accepted, and the datapoint of the corresponding participants marked by a white instead of a black outline (see Fig. 2, *E*–*H*).

#### Data exclusion.

We excluded trials from analysis using the following criteria: the vertical amplitude of the primary saccade covered less than 50% of the distance to the eye movement target, the absolute horizontal amplitude of the primary saccade was larger than 6° (i.e., the primary saccade likely targeted something other than the eye movement target or a candidate stimulus), participants blinked between eye movement target onset and onset of the first candidate stimulus, no primary saccade was detected up to 1,500 ms after eye movement target onset, gaze deviated more than 2° from the fixation cross in a time window from –20 to 80 ms relative to eye movement target onset, and when no perceptual judgement was provided within a time-frame of 1,500 ms after response phase onset (only adaptation trials). In addition, some trials had to be excluded due to technical difficulties during data recording.

In the small-target experiment, an average of *M* = 11.95% of trials in the discriminate-1st condition (min = 1.71%, max = 32.29%), *M* = 10.76% of trials in the discriminate-2nd condition (min = 0.86%, max = 37.71%), *M* = 9.81% of trials in the discriminate-1st-no-2nd condition (min = 0.86%, max = 28.00%), and *M* = 13.63% of trials in the discriminate-2nd-no-1st condition (min = 2.00%, max = 33.14%) had to be excluded. In the large-target experiment, an average of *M* = 10.35% of trials in the discriminate-1st condition (min = 1.14%, max = 23.43%), *M* = 11.07% of trials in the discriminate-2nd condition (min = 0.86%, max = 31.43%), *M* = 12.51% of trials in the discriminate-1st-no-2nd condition (min = 3.14%, max = 26.57%), and *M* = 10.05% of trials in the discriminate-2nd-no-1st condition (min = 0.57%, max = 28.86%) had to be excluded.

### Modeling

To estimate the relative contribution of different learning processes to motor learning, as observed in our paradigm, we fitted a state-space model to the data of each individual participant (23, 24). Our model assumes that the horizontal saccade amplitude component in each trial *n* (horAmp*_n_*) corresponds to the weighted (*w*) sum of the state of a strategic learning process in trial *n* (strategic*_n_*) as well as the state of a gradual learning process in trial *n* (gradual_*n*_)

(*1*) $${\text{horAmp}}_{n}=\left(w\times {\text{strategic}}_{n}\right)+[\left(1-w\right)\times {\text{gradual}}_{n}]$$

Here, horAmp is the output of our model, and the free parameter *w* quantifies the relative contribution of the strategic and the gradual process to the predicted saccade amplitude. For example, if *w* equals unity, the predicted saccade amplitude would be entirely driven by the strategic process, which would result in a step-function like predicted adaptation time course. Conversely, if *w* equals zero, the predicted saccade amplitude would be entirely driven by the gradual process. Intermediate values of *w* imply that both processes contribute to some extent to the predicted saccade amplitude.

The state of the strategic learning process is updated for each trial by compensating the full error between the location of the task-relevant candidate stimulus (loc) and the saccade amplitude in the previous trial *n* – 1. Thus, whenever a movement error is experienced in a given trial *n −* 1, the strategic learning process compensates fully for the experienced error, so that the saccade in the subsequent trial *n* is perfectly accurate, i.e., it lands exactly on the location of the task-relevant candidate stimulus, yielding a movement error of zero in trial *n*

(*2*) $${\text{strategic}}_{n}={\text{horAmp}}_{n-1}-[{\text{horAmp}}_{n-1}-({\text{loc}}_{n-1}\times d)]$$

The strategic learning process quantifies any voluntary strategy participants might use to explicitly target saccadic eye movements. This process is similar to the explicit learning process, typically found to contribute to adaptation of reaching movements (e.g., see Ref. 40). Since some participants showed saccade adaptation to the location of the task-irrelevant stimulus (e.g., Fig. 2*B*), we introduced a free parameter *d*. The free parameter is binary (either −1 or 1), and controls if a participant chooses the task-relevant (*d* = 1) or the task-irrelevant (*d* = −1; i.e., the second candidate stimulus in the discriminate-1st conditions, and the first candidate stimulus in the discriminate-2nd conditions) candidate stimulus as adaptation target.

The state of the gradual learning process is also updated for each trial, by compensating a proportion *r* of the movement error, experienced in the previous trial *n −* 1

(*3*) $${\text{gradual}}_{n}={\text{horAmp}}_{n-1}-r\times [{\text{horAmp}}_{n-1}-({\text{loc}}_{n-1}\times d)]$$

Thus, here, *r* corresponds to the learning rate, and the gradual learning process itself captures the gradual trial-to-trial changes in saccade amplitude, typically observed in double-step saccade adaptation paradigms (e.g., see Refs. 37 and 41).

The location of the task-relevant candidate stimulus (loc) was set to zero in baseline trials since no candidate stimulus was shown in that phase. The model was initialized with a prediction of the horizontal saccade amplitude in the first trial, based on a participant’s average horizontal amplitude in the baseline phase. To stabilize model fits, we repeated the fitting procedure 101 times for each participant, each time using different starting values for the free parameter *r*. The best-fitting result of this procedure [evaluated using the Bayesian information criterion (BIC) (42)] was then used as the final model output. Since we were primarily interested in quantifying the relative contribution of strategic adjustment and gradual learning to amplitude changes during saccade adaptation, we fitted the model only to trials from baseline and adaptation trials, but not retention trials.

## RESULTS

We investigated the evaluation of temporally dynamic and competing error signals in saccade adaptation by presenting two postsaccadic candidate stimuli that were spatially and temporally offset. In two experiments, we varied the properties of the saccade target, and in four conditions per experiment, we varied the task-relevance and presence of the first and second candidate stimulus.

### Obligatory Use of Early Postsaccadic Error Signals in the Small-Target Experiment

Generally, most participants across all four conditions of the small-target experiment showed an above-chance discrimination performance, which decreased with increasing retinal error relative to the location of the respective task-relevant candidate stimulus (Supplemental Fig. S1, *A*–*D*; all Supplemental material is available at https://doi.org/10.5281/zenodo.7128738). Thus, spatially accurate saccades (i.e., saccades that ended close to the position of the respective task-relevant candidate stimulus) were beneficial for task performance across all conditions of the small-target experiment.

To quantify which error signal was used by the oculomotor system to choose a target for saccade adaptation in the four conditions of the small-target experiment, we calculated the average horizontal saccade amplitude in the last block of the respective adaptation trials. We observed, across all conditions of the small-target experiment, a steep change in the horizontal primary saccade amplitude shortly after we introduced the perceptual task in the respective adaptation phase (Fig. 2, *A*–*D*). In the discriminate-1st condition, where two candidate stimuli were shown sequentially and participants had to discriminate a feature on the first stimulus, horizontal saccade amplitudes notably increased by the end of the adaptation phase [*M* = 1.08°, 95% confidence interval (CI_95%_) = [0.95°, 1.21°] (Fig. 2, *A* and *E*)]. The magnitude of this increase was comparable with the amplitude change in the discriminate-1st-no-2nd condition, where only the first candidate stimulus was shown upon saccade offset [*M* = 1.05°, CI_95%_ = [0.95°, 1.16°] (Fig. 2, *C* and *G*)].

A similar change in the horizontal saccade amplitude was observed at the end of the adaptation phase in the discriminate-2nd condition, where two candidate stimuli were shown sequentially, and participants had to discriminate a feature on the second candidate [*M* = 0.51°, CI_95%_ = [0.24°, 0.77°] (Fig. 2, *B* and *F*)]. However, the amplitude changes in the discriminate-2nd condition had a smaller magnitude and a different sign compared with the amplitude changes in the discriminate-2nd-no-1st condition, where only the second candidate stimulus was shown ∼350 ms after saccade offset [*M* = –0.71°, CI_95%_ = [–1.00°, –0.42°] (Fig. 2, *D* and *H*)]. Critically, whereas amplitude changes in the discriminate-1st, discriminate-1st-no-2nd, and discriminate-2nd-no-1st condition were directed toward the location of the respective task-relevant candidate stimulus, the amplitude change in the discriminate-2nd condition was mostly directed toward the location of the task-irrelevant, first candidate stimulus. More specifically, the sign of individual mean amplitude changes revealed that most participants in the discriminate-2nd condition adapted toward the location of the task-irrelevant candidate stimulus (18 out of 21 participants with significant amplitude changes had a positive sign), whereas participants in the discriminate-2nd-no-1st condition mostly adapted toward the location of the task-relevant candidate stimulus (18 of 22 participants with significant amplitude changes had a negative sign).

This failure to suppress the error signal from the first, task-irrelevant candidate stimulus was not due to the oculomotor system generally ignoring delayed error signals: We observed robust saccade adaptation in a control condition, where the only available error signal came from a candidate stimulus that was shown ∼350 ms after saccade offset (Fig. 2, *D* and *H*). Furthermore, the observed failure to suppress the error signal from the first, task-irrelevant candidate stimulus was also not due to participants misunderstanding the task: Task-performance in the discriminate-2nd condition of the small-target experiment was above chance (Supplemental Fig. S1*B*), and primary saccades in this condition were often followed by additional secondary saccades toward the location of the second, task-relevant candidate stimulus (Supplemental Fig. S2, *B* and *F*). This suggests that participants knew which stimulus they had to discriminate, but the oculomotor system failed to utilize the right error signal from the second, task-relevant candidate stimulus.

One hallmark of saccade adaptation is that adaptive changes to saccade amplitudes typically persist for some time, even in the absence of experimental perturbations, and that participants have to actively unlearn the acquired eye movement behavior in the absence of experimental perturbations to eye movement accuracy (43). To check the persistence of amplitude changes in our paradigm, we calculated the horizontal saccade amplitude in the first block of retention trials, where no candidate stimuli were shown. Indeed, we found that horizontal saccade amplitudes across all conditions of the small-target experiment did not immediately return to baseline once participants were no longer required to complete a perceptual task after the primary saccade. Instead, adapted horizontal saccade amplitudes from the previous block of adaptation trials persisted to some degree in the subsequent retention trials [discriminate-1st: *M* = 0.72°, CI_95%_ = [0.60°, 0.85°] (Fig. 2, *A* and *E*); discriminate-2nd: *M* = 0.57°, CI_95%_ = [0.39°, 0.76°] (Fig. 2, *B* and *F*); discriminate-1st-no-2nd: *M* = 0.78°, CI_95%_ = [0.67°, 0.90°] (Fig. 2, *C* and *G*)]; discriminate-2nd-no-1st: *M* = –0.28°, CI_95%_ = [–0.45°, –0.10°] (Fig. 2, *D* and *H*)]. Whereas most participants in the discriminate-2nd condition showed retention (23 of 24 participants with significant amplitude changes), this effect was slightly more variable in the discriminate-2nd-no-1st condition (18 of 24 participants with significant amplitude changes). Amplitude changes in both adaptation (Supplemental Table S1) and retention (Supplemental Table S2) trials of the discriminate-1st, discriminate-2nd, and discriminate-1st-no-2nd condition were positively correlated. This, on the one hand, suggests stable interindividual differences across conditions of the small-target experiment, and, on the other hand, highlights the similarity of the adaptation effect, observed across conditions of this experiment.

To summarize, we found that saccade adaptation in the small-target experiment prioritized the error signal from a task-relevant candidate stimulus, while suppressing the error signal of a task-irrelevant candidate stimulus, whenever the task-relevant error signal was available immediately after saccade offset (discriminate-1st condition). However, we found no such prioritization when the error signal from a task-relevant candidate stimulus only became available ∼350 ms after saccade offset, while competing with the error signal from a task-irrelevant candidate stimulus, shown upon saccade offset. Instead, saccade adaptation utilized whatever error signal was available immediately upon saccade offset, even if this resulted in amplitude changes toward the task-irrelevant candidate stimulus (discriminate-2nd condition).

### Eye Movement Target Properties Modulate Prioritization of Error Signals

In a previous study (30), we used a large ring as eye movement target, and found that saccade adaptation selectively processed the error signal from a task-relevant stimulus, while suppressing the error signal from a simultaneously shown task-irrelevant stimulus. Thus, one reason for why saccade adaptation utilized the error signal of the task-irrelevant candidate stimulus in the discriminate-2nd condition of the small-target experiment might be related to properties of the eye movement target we have used. To test this, we designed the large-target experiment, where a large ring was used as eye movement target, similar to our previous study (30, see also Ref. 44).

Similar to the small-target experiment, participants showed an above-chance perceptual performance across all conditions of the large-target experiment, and perceptual performance decreased with increasing retinal error relative to the location of the task-relevant candidate stimulus (Supplemental Fig. S1, *E–H*). As in the small-target experiment, we observed a steep change in the horizontal saccade amplitude once we introduced a perceptual task in adaptation trials of the large-target experiment (Fig. 2). In the discriminate-1st condition of the large-target experiment, saccade amplitudes notably increased by the end of the adaptation phase [*M* = 1.46°, CI_95%_ = [1.28°, 1.65°] (Fig. 2, *A* and *E*)], and the magnitude of the observed change was, once again, comparable with the change in the discriminate-1st-no-2nd condition [*M* = 1.45°, CI_95%_ = [1.28°, 1.62°] (Fig. 2, *C* and *G*)]. However, although horizontal saccade amplitudes notably decreased by the end of the adaptation phase in the discriminate-2nd-no-1st condition [*M* = –1.04°, CI_95%_ = [–1.24°, –0.85°] (Fig. 2, *D* and *H*)], we observed no such change in the discriminate-2nd condition of the large-target experiment [*M* = −0.38°, CI_95%_ = [−0.80°, 0.04°] (Fig. 2, *B* and *F*)]. Critically, the absence of a group effect in the discriminate-2nd condition was not due to individual participants showing no amplitude changes (22 of 24 participants with significant amplitude changes), but because participants adapted inconsistently: whereas some participants in the discriminate-2nd condition of the large-target experiment showed amplitude changes toward the first, task-irrelevant candidate stimulus (8 of 22 participants with significant amplitude changes had a positive sign), other participants showed amplitude changes toward the second, task-relevant candidate stimulus (14 of 22 participants with significant amplitude changes had a negative sign). Primary saccades in the large-target experiment were often followed by additional secondary saccades, which compensated for any residual movement error, and brought gaze close to the location of the respective task-relevant candidate stimulus. Although such secondary saccades were observable across all conditions of the large-target experiment, they were especially pronounced in conditions where the second candidate stimulus was task-relevant (Supplemental Fig. S2, *A–D* and *I–L*). A robust 2×4 mixed ANOVA with the horizontal primary saccade amplitudes in the last block of adaptation trials as its dependent variable showed a significant main effect of the factor condition (discriminate-1st, discriminate-2nd, discriminate-1st-no-2nd, discriminate-2nd-no-1st), *F*(3,21.95) = 149.79, *P* < 0.001, and a significant interaction between the factors experiment (small-target, large-target) and condition, *F*(3,21.95) = 10.89, *P* < 0.001. However, we found no significant main effect of the factor experiment, *F*(1,28.92) = 0.61, *P* = 0.442.

Similar to the small-target experiment, changes to horizontal saccade amplitudes that were acquired in adaptation trials of the large-target experiment persisted to some degree even in the subsequent retention trials [discriminate-1st: *M* = 0.50°, CI_95%_ = [0.40°, 0.60°] (Fig. 2, *A* and *E*); discriminate-1st-no-2nd: *M* = 0.57°, CI_95%_ = [0.40°, 0.73°] (Fig. 2, *C* and *G*); discriminate-2nd-no-1st condition: *M* = –0.49°, CI_95%_ = [–0.62°, –0.35°] (Fig. 2, *D* and *H*)]. The only exception to this was the discriminate-2nd condition, where horizontal saccade amplitudes returned close to their baseline value in the first block of retention trials [*M* = –0.09°, CI_95%_ = [–0.23°, 0.06°] (Fig. 2, *B* and *F*)]. The absence of a group effect in the discriminate-2nd condition could, once again, not be explained by participants not adapting their horizontal saccade amplitudes (18 of 24 participants with significant amplitude changes), but its absence was instead due to individual differences in adaptation direction, as observed in adaptation trials. A robust 2×4 mixed ANOVA showed significant main effects of the factors experiment, *F*(1,29.95) = 39.93, *P* < 0.001, and condition, *F*(3,23.73) = 73.01, *P* < 0.001, as well as a significant interaction between those two factors, *F*(3,23.73) = 3.91, *P* = 0.021. Similar to the small-target experiment, amplitude changes in adaptation (Supplemental Table S1) and retention trials (Supplemental Table S2) of the discriminate-1st, and discriminate-1st-no-2nd condition were positively correlated.

To summarize, unlike in the small-target experiment, saccade adaptation in the large-target experiment successfully suppressed error signals from task-irrelevant candidate stimuli, irrespective of whether the error signal from the task-irrelevant stimulus was available immediately after saccade offset (discriminate-1st condition) or after a temporal delay relative to saccade offset (discriminate-2nd condition). However, this suppression of task-irrelevant error signals did not always come along with a prioritization of the error signal from a task-relevant candidate stimulus: Although horizontal saccade amplitudes of participants showed a bias toward the task-relevant candidate stimulus in the discriminate-2nd condition of the large-target experiment, this bias disappeared rapidly once participants no longer had to complete a perceptual task. Data of individual participants suggests that this was not due to an absence of significant saccade adaptation, but due to inconsistencies in which candidate was chosen as adaptation target.

### Strategic and Gradual Adjustments Contribute to Oculomotor Learning

Two differences between the adaptation time courses in the small-target and large-target experiment are notable. First, horizontal saccade amplitudes across all conditions of the small-target experiment showed a steep change at the onset of the respective adaptation phase. This initial steep correction was followed by subsequent gradual changes to horizontal saccade amplitudes throughout the rest of the adaptation phase, bringing gaze gradually closer to whatever was chosen as target for saccade adaptation (Fig. 2, *A*–*D*). Such gradual changes are characteristic for saccade adaptation (43), and appear to be less pronounced in the large-target experiment. From a modeling perspective, those gradual changes are often interpreted as the consequence of a gradual learning process that compensates for a fraction of the experienced motor error in each trial (e.g., see Ref. 37). Second, adaptive amplitude changes in the small-target experiment persisted to some degree even when participants did not have to complete a perceptual task in retention trials. Such after effects are, again, characteristic for saccade adaptation (43), and they were also observable in most conditions of the large-target experiment. The only exception to this is the discriminate-2nd condition of the large-target experiment, where amplitude changes, at the group level, disappeared at the onset of retention trials, and where the average adaptation time course resembles a step-function.

One reason for those differences might be that saccade adaptation in our paradigm is driven by both, a gradual learning process as well as a strategic adjustment of eye movement behavior to the requirements of our perceptual task (23, 45). The contribution of those two processes might differ between our two experiments, so that strategic adjustment dominates amplitude changes in some instances, resulting in uncharacteristic, step-function-like time courses. To test for this, we used a state-space model, proposed by Schütz et al. (23), and fitted it to the data of individual participants from both of our experiments. The model assumes that the horizontal saccade amplitude in each trial is the consequence of two processes: one process quantifies the strategic adjustment of eye movement behavior to task requirements, whereas a second process quantifies the contribution of a gradual learning process that compensates for a fraction of the movement error, which was experienced in a trial.

**Figure 3.**
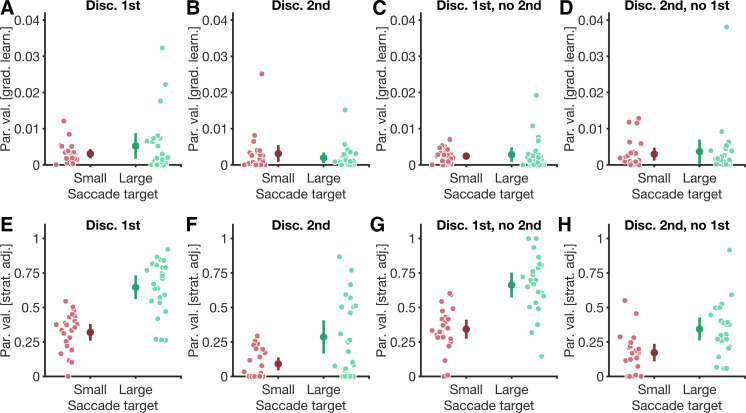
Model parameters. Values of parameters (Par. val.), quantifying the contribution of the gradual learning process (grad. learn.; *A*–*D*) and the immediate strategic adjustment (strat. adj.; *E*–*H*) in the four conditions (Discriminate-1st, Discriminate-2nd, Discriminate-1st-no-2nd, Discriminate-2nd-no-1st) of the small-target (red datapoints) and large-target experiment (green datapoints). *A*–*H*: large datapoints are averages across participants, small datapoints are data of individual participants. Error bars are 95% confidence intervals. Note the different scaling of the *y*-axis in *E*–*H* compared with *A*–*D*.

Our model showed a good fit to the data from baseline as well as adaptation trials of all conditions in both experiments (Fig. 2, *A*–*D*). Inspecting the model parameters revealed that gradual learning (i.e., the *r* parameter in Eq. *3*) compensated for a comparable percentage of the retinal error in trials of the small-target experiment [discriminate-1st: *M* = 0.31%, CI_95%_ = [0.19%, 0.42%], (Fig. 3*A*); discriminate-2nd: *M* = 0.31%, CI_95%_ = [0.09%, 0.53%], (Fig. 3*B*); discriminate-1st-no-2nd: *M* = 0.24%, CI_95%_ = [0.16%, 0.32%], (Fig. 3*C*); discriminate-2nd-no-1st: *M* = 0.30%, CI_95%_ = [0.13%, 0.46%], (Fig. 3*D*)], and large-target experiment [discriminate-1st: *M* = 0.52%, CI_95%_ = [0.18%, 0.86%], (Fig. 3*A*); discriminate-2nd: *M* = 0.20%, CI_95%_ = [0.06%, 0.33%], (Fig. 3*B*); discriminate-1st-no-2nd: *M* = 0.28%, CI_95%_ = [0.10%, 0.47%], (Fig. 3*C*); discriminate-2nd-no-1st: *M* = 0.37%, CI_95%_ = [0.04%, 0.69%], (Fig. 3*D*)]. Indeed, a robust 2×4 mixed ANOVA showed no significant main effects of the factors condition, *F*(3,23.05) = 1.20, *P* = 0.333, and experiment, *F*(1,29.69) = 0.48, *P* = 0.494, and no significant interaction between the factors, *F*(3,23.05) = 0.36, *P* = 0.784.

Inspecting the strategic adjustment parameter revealed that the parameter had a smaller value across all conditions of the small-target experiment [discriminate-1st: *M* = 31.97%, CI_95%_ = [26.23%, 37.72%], (Fig. 3*E*); discriminate-2nd: *M* = 09.04%, CI_95%_ = [04.67%, 13.40%], (Fig. 3*F*); discriminate-1st-no-2nd: *M* = 34.22%, CI_95%_ = [27.65%, 40.78%], (Fig. 3*G*); discriminate-2nd-no-1st condition: *M* = 17.24%, CI_95%_ = [11.25%, 23.24%], (Fig. 3*H*)], compared with conditions in the large-target experiment [discriminate-1st: *M* = 64.59%, CI_95%_ = [56.32%, 72.86%], (Fig. 3*E*); discriminate-2nd: *M* = 28.58%, CI_95%_ = [16.85%, 40.31%], (Fig. 3*F*); discriminate-1st-no-2nd: *M* = 66.27%, CI_95%_ = [57.56%, 74.99%], (Fig. 3*G*); discriminate-2nd-no-1st condition: *M* = 34.34%, CI_95%_ = [26.31%, 42.37%], (Fig. 3*H*)]. A robust 2×4 mixed ANOVA showed significant main effects of the factors condition, *F*(3,22.49) = 28.33, *P* < 0.001, and experiment, *F*(1,21.14) = 63.44, *P* < 0.001, but no significant interaction between the factors, *F*(3,22.49) = 1.93, *P* = 0.154.

To summarize, results of model fitting revealed that eye movement target properties not only influenced which error signal saccade adaptation used to evaluate movement accuracy, but eye movement target properties also modulated how saccade adaptation corrects perturbations to movement accuracy. More specifically, although a strategic component contributed to all conditions across both experiments, the contribution of strategic adjustment was stronger in the large-target compared with the small-target experiment. However, despite the contribution of a strategic component in the large-target experiment, model fits also revealed that gradual learning was present in this experiment, and thus, the observed amplitude changes cannot be fully explained by participants strategically targeting specific on-screen locations.

### Saccade Adaptation Has a Differential Influence on Early and Late Parts of Saccade Trajectories

Saccade trajectories are not uniformly affected by saccade adaptation. For example, saccade trajectories become curved over the course of saccade adaptation, and curvature develops slower for early parts of saccade trajectories, compared with later trajectory parts (36). A similar differential effect was recently observed for the spatial displacement during the acceleration and deceleration phase of saccades, with spatial displacement during saccade deceleration being stronger affected by saccade adaptation, compared with spatial displacement during saccade acceleration (38). Critically, this effect was absent when participants were instructed to strategically target a specific on-screen location. Thus, if the observed amplitude changes in our two experiments are the consequence of oculomotor learning, and not just an expression of participants strategically targeting the location where they expect the task-relevant candidate stimulus to appear, we would expect to observe a similar differential effect on saccade acceleration and deceleration during adaptation trials.

**Figure 4.**
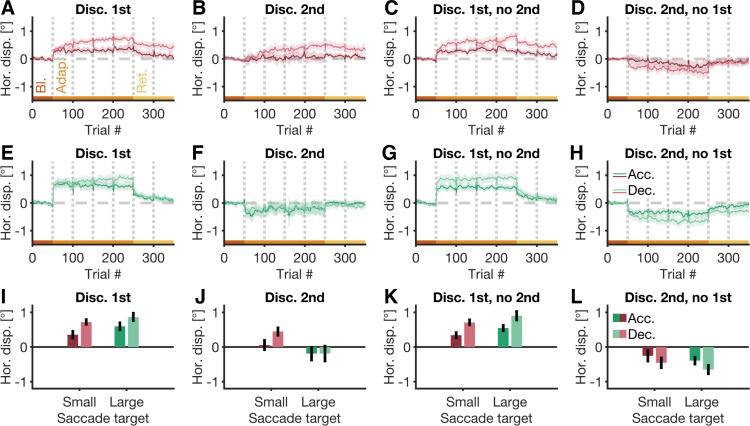
Horizontal displacement in the acceleration and deceleration phase of primary saccade traces. *A*–*H*: horizontal displacement (Hor. disp.) in the acceleration (Acc.; dark colors) and deceleration phase of saccade traces (Dec.; bright colors) in the four conditions (Discriminate-1st, Discriminate-2nd, Discriminate-1st-no-2nd, Discriminate-2nd-no-1st) of the small-target (red) and large-target experiment (green). Thin colored lines represent moving averages over the respective horizontal displacement. Dotted gray lines are set breaks. The shaded areas around the moving averages are 95% confidence intervals of the respective moving average. Data is shown for baseline (Bl.), adaptation (Adap.), and retention (Ret.) trials. *I*–*L*: average horizontal displacement in the last block of adaptation trials, separately for the acceleration (dark colors) and deceleration phase (bright colors) of primary saccade traces in the four conditions of the small-target (red) and large-target experiment (green). Error bars are 95% confidence intervals.

To test for this, we calculated, for each primary saccade separately, the respective peak velocity, and used it to divide a saccade’s horizontal trace into an early (i.e., horizontal saccade trace before peak velocity) and a late part (i.e., horizontal saccade trace after peak velocity). Calculating the horizontal saccade amplitude for each of those two phases yields the horizontal displacement during the saccade’s acceleration phase (i.e., it’s early part) and deceleration phase (i.e., it’s late part), respectively. Similar to horizontal saccade amplitudes (Fig. 2), we observed a change in the horizontal displacement during saccade acceleration and deceleration at the onset of adaptation trials in most conditions of both experiments (Fig. 4). By the end of the adaptation phase in the small-target experiment, both, horizontal displacement during the saccade’s acceleration and deceleration phase, notably changed, and this change was more pronounced in the deceleration phase (discriminate-1st: *M* = 0.72°, CI_95%_ = [0.63°, 0.82°], Fig. 4, *A* and *I*; discriminate-1st-no-2nd: *M* = 0.71°, CI_95%_ = [0.61°, 0.81°], Fig. 4, *C* and *K*; discriminate-2nd-no-1st condition: *M* = −0.46°, CI_95%_ = [–0.62°, –0.29°], Fig. 4, *D* and *L*), compared with the acceleration phase (discriminate-1st: *M* = 0.36°, CI_95%_ = [0.23°, 0.48°], Fig. 4, *A* and *I*; discriminate-1st-no-2nd: *M* = 0.34°, CI_95%_ = [0.24°, 0.44°], Fig. 4, *C* and *K*; discriminate-2nd-no-1st condition: *M* = −0.25°, CI_95%_ = [–0.43°, –0.07°], Fig. 4, *D* and *L*). Notably, the horizontal displacement during the acceleration phase in the discriminate-2nd condition of the small-target experiment was close to zero (*M* = 0.06°, CI_95%_ = [–0.10°, 0.22°], Fig. 4, *B* and *J*), whereas the horizontal displacement during the deceleration phase increased by a similar magnitude as in the other conditions of this experiment (*M* = 0.45°, CI_95%_ = [0.32°, 0.58°], Fig. 4, *B* and *J*).

We observed a similar pattern in the large-target experiment, with horizontal displacement, depending on the experimental condition, either notably increasing or decreasing by the end of the respective adaptation phase. For most conditions in the large-target experiment, this change in horizontal displacement was more pronounced for the deceleration phase of saccades (discriminate-1st: *M* = 0.87°, CI_95%_ = [0.73°, 1.00°], Fig. 4, *E* and *I*; discriminate-1st-no-2nd: *M* = 0.90°, CI_95%_ = [0.76°, 1.05°], Fig. 4, *G* and *K*; discriminate-2nd-no-1st condition: *M* = –0.65°, CI_95%_ = [–0.80°, –0.50°], Fig. 4, *H* and *L*), compared with their acceleration phase (discriminate-1st: *M* = 0.60°, CI_95%_ = [0.47°, 0.72°], Fig. 4, *E* and *I*; discriminate-1st-no-2nd: *M* = 0.55°, CI_95%_ = [0.45°, 0.65°], Fig. 4, *G* and *K*; discriminate-2nd-no-1st condition: *M* = –0.39°, CI_95%_ = [–0.52°, –0.27°], Fig. 4, *H* and *L*). The only exception to this pattern was the discriminate-2nd condition, where horizontal displacement by the end of the respective adaptation phase was similar for the acceleration (*M* = –0.19°, CI_95%_ = [–0.40°, 0.02°], Fig. 4, *F* and *J*) and deceleration phase (*M* = −0.19°, CI_95%_ = [–0.43°, 0.05°], Fig. 4, *F* and *J*). A robust 2×4 mixed ANOVA, with the delta between the horizontal displacement in the acceleration and deceleration phase in the last block of adaptation trials as dependent variable, showed a significant main effect of the factor experiment, *F*(1,29.56) = 6.33, *P* = 0.018, a significant main effect of the factor condition, *F*(3,24.23) = 13.35, *P* < 0.001, and a significant interaction between the factors, *F*(3,24.23) = 3.17, *P* = 0.043.

To summarize, we found that saccade adaption in our paradigm had, similar to earlier findings (38), a differential effect on early and late parts of saccade trajectories. Critically, we observed this differential effect in both, the small-target and large-target experiment, despite the large contribution of strategic adjustment to empirical amplitude changes, especially in the large-target experiment. Thus, the observed amplitude changes in most conditions of both experiments are most likely the consequence of genuine oculomotor learning, and not just a mere strategic adjustment of eye movements to task-requirements.

## DISCUSSION

In two experiments, we investigated how saccade adaptation evaluates eye movement accuracy in dynamic environments, where the availability of two competing error signals varied over time. We found that properties of the eye movement target, used to elicit a primary saccade, have a profound influence on various aspects of oculomotor learning, as observed in our paradigm.

### Eye Movement Target Properties Modulate Target Selection for Saccade Adaptation

When using a small, filled square as eye movement target, we observed that saccade adaptation utilized the error signal from a task-relevant candidate stimulus, shown upon saccade offset, while selectively suppressing the error signal from a task-irrelevant candidate stimulus, shown ∼350 ms after saccade termination (Fig. 2, *A* and *E*). However, when the task-irrelevant candidate stimulus was shown upon saccade offset, and its error signal was, thus, the first available error signal after the eye movement, saccade adaptation evaluated movement accuracy based on the task-irrelevant error signal, even though this resulted in saccade adaptation away from the delayed, task-relevant visual information (Fig. 2, *B* and *F*). This pattern was markedly different when we used a large ring as eye movement target. Here, saccade adaptation, on the one hand, successfully suppressed the task-irrelevant error signal, irrespective of the timing of its availability. However, on the other hand, the task-relevant error signal was not always used to adapt horizontal saccade amplitudes in a lasting manner (Fig. 2, *A, B* and *E, F*).

This difference between which error signal was utilized in the discriminate-2nd conditions of the small-target and large-target experiment might be related to two different aspects of the stimuli: first, the size of the presaccadic locus of attention and second, the similarity of the presaccadic target and the postsaccadic candidate stimulus. The first hypothesis attributes the differential results to differences in the size of the presaccadic locus of attention. The eye movement target in the small-target experiment promoted a relatively small locus of presaccadic attention, limited to the area of the small eye movement target. The sudden appearance of the first candidate stimulus in the discriminate-2nd condition of the small-target experiment might, thus, have constituted a salient event for the oculomotor system, which resulted in an involuntary and obligatory postsaccadic attention shift toward the location of whichever candidate was first presented after the saccade (16, see also Refs. 22 and 25). Consequently, movement accuracy was evaluated based on the error signal from the first shown candidate stimulus, and the corresponding candidate stimulus was chosen as target for saccade adaptation, irrespective of its actual task-relevance. This interpretation is also in line with findings on adaptation of reaching movements, where a sensory prediction error was found to induce adaptation, even though this interfered with task-demands (18, 46).

The eye movement target in the large-target experiment, however, promoted a relatively large locus of presaccadic attention, allocated across the entire area that was surrounded by the large ring. Consequently, both candidate stimuli automatically fell into the postsaccadic locus of attention, which might have suppressed obligatory attention shifts, and allowed for a greater flexibility in selecting which error signal to use for evaluation of saccade accuracy. This interpretation is in line with other findings on the coupling between adaptation of reactive saccades and attention (47, 48), on the relevance of attention shifts in visual working memory for target selection in delayed saccade adaptation (30), and on the relationship between saccade latency and the scale of the attentional locus (49–52). The latter studies demonstrated that latencies of saccades toward a large, unfilled ring are delayed, compared with saccades toward a smaller ring (49–52). One potential interpretation for this size-latency effect are the lower perceptual costs that saccades toward a large eye movement target carry, since the perceptual costs of a movement error in the presence of a large saccade target are less detrimental, compared with when a small saccade target is used (see also Refs. 53 and 54). Similarly, the perceptual costs following inaccurate saccades in our large-target experiment might be less severe compared with the perceptual costs following inaccurate saccade in the small-target experiment, and this might have contributed to the differences observed between our two experiments.

However, despite this supposedly greater flexibility in selecting which error signal to use for evaluation of saccade accuracy, we found no consistent saccade adaptation in the discriminate-2nd condition of the large-target experiment (Fig. 2, *B* and *F*). Instead, horizontal saccade amplitudes on the group level showed a small bias toward the location of the respective task-relevant candidate stimulus, and this bias disappeared entirely in retention trials, where no candidate stimuli where shown. The absence of a group effect in this condition was due to individual participants adapting inconsistently, with some participants adapting toward, whereas others adapted away from the task-relevant candidate stimulus. Irrespective of the adaptation direction of the first saccade, participants made frequent use of additional secondary saccades to bring gaze close to the location of the task-relevant candidate stimulus (Supplemental Fig. S2, *B* and *J*).

The second hypothesis attributes adaptation differences between the small- and large-target experiments to differences in the similarity of the pre- and postsaccadic stimuli. The obligatory adaptation to the first candidate stimulus in the small-target experiment might be due to the visual similarity between the filled square and the two outlined squares, which were used as eye movement target and candidate stimuli. Indeed, previous research found evidence that shifting the position of an eye movement target while a saccade is in flight, and simultaneously showing a differently looking distractor at the saccade target’s former presaccadic position, leads to saccade adaptation toward the eye movement target’s postsaccadic position (17). This result implies that saccade adaptation is sensitive to the visual object identity of the eye movement target and uses it to track the target’s location before and after a saccade (but see Ref. 55).

Overall, based on the results of our two experiments and findings from previous studies, we would consider the attentional locus hypothesis to be the more likely explanation for the differences between the discriminate-2nd conditions of the small-target and large-target experiment. For example, Ditterich et al. (44), found evidence that information from perisaccadic shifts of a background image, on which a saccade target is placed, is only used for evaluation of saccade accuracy when a large, unfilled ring is used as saccade target, but not when a small cross constitutes the saccade target. This result suggests that the oculomotor system interprets everything that falls within the area of the circular saccade target automatically as being part of the eye movement target, resulting in a contribution of the corresponding information to saccade accuracy evaluation. Since we used a large, unfilled ring as saccade target in the large-target experiment, it is likely that the oculomotor system interpreted the two postsaccadic candidate stimuli as part of the saccade target, which would make the similarity hypothesis unlikely. However, based on our data we cannot definitely rule out one or the other hypothesis for why obligatory adaptation toward the first candidate stimulus occurred, and additional follow-up experiments are required to disambiguate those two hypotheses.

It is important to note, however, that the large-target and small-target experiment not only differed regarding the properties of the respective eye movement target, but also regarding the exact stimulus that was shown after a primary saccade. More precisely, whereas candidate stimuli were shown together with a large, unfilled ring in conditions of the large-target experiment, no ring was presented in conditions of the small-target experiment. Instead, participants in the small-target experiment only saw the two candidate stimuli after a primary saccade. In adaptation trials of the large-target experiment, the presence of the large, unfilled ring might have encouraged a competition between the locations of the two candidate stimuli, which, in turn, could have influenced properties of oculomotor learning in this experiment. Furthermore, the large, unfilled ring was also shown in baseline and adaptation trials of the large-target experiment, whereas participants saw an empty gray screen in adaptation and baseline trials of the small-target experiment. The presence of the ring in adaptation and baseline trials of the large-target experiment might have generated an additional error signal, which affected oculomotor learning differently compared with the corresponding trials in conditions of the small-target experiment. However, since learning rates of the gradual learning process, as estimated by our model, were similar across experiments, we believe that the later alternative explanation is unlikely.

### Eye Movement Target Properties Modulate Which Learning Processes Contribute to Saccade Adaptation

The properties of the eye movement target not only influenced which error signal was used by saccade adaptation to evaluate movement accuracy, but they also affected how saccades were corrected. Across all conditions of both experiments, saccade adaptation was driven by the contribution of two distinct processes: A gradual learning process compensated for a fraction of the movement error in a trial, and a voluntary process was deployed to strategically adjust saccade amplitudes to the requirements of our perceptual task. However, the contribution of these processes differed between experiments (Fig. 3). This resulted in markedly different adaptation time courses in the large-target compared with the small-target experiment (Fig. 2).

This difference between adaptation time courses in our two experiments might be related to the stronger contribution of the strategic process in the large-target compared with the small-target experiment, and the stronger contribution of the strategic process might have been a consequence of the properties of the large ring, used as eye movement target. More specifically, the large ring, used as eye movement target in the large-target experiment, might have served as a landmark, which allowed for an easier spatial localization of the task-relevant candidate stimulus after a saccade. Participants in the large-target experiment might have, thus, relied more strongly on internal predictions about the future location of the task-relevant candidate stimulus, and used those predictions to strategically target saccades toward where they expect the task-relevant candidate stimulus to appear. Given the negative relationship between postsaccadic retinal error and discrimination performance (Supplemental Fig. S1), this strategy is beneficial for task-performance, and would allow participants to achieve a higher performance earlier in the adaptation phase. Since the eye movement target in the small-target experiment provided no landmark (i.e., it disappeared upon saccade onset), participants in conditions of this experiments likely relied to a lesser extent on external landmarks (and a strategic process), when targeting saccades. As an alternative to the landmark hypothesis, the large ring might also have facilitated the strategic process because its visual signal is spatially less constrained and uncertain, compared with the visual signal from the small target. Consequently, it might be easier to modulate the ultimate saccade end point by top-down guidance toward the expected location of the task-relevant candidate stimulus. Further experiments would be necessary to disentangle the landmark and the position uncertainty hypothesis.

In the literature, saccade adaptation is typically described as being the consequence of a gradual learning process, which progresses involuntarily and without awareness of participants (e.g., Ref. 26). The contribution of a strategic process might thus be interpreted as evidence that the observed amplitude changes in our paradigm are not due to oculomotor learning (i.e., saccade adaptation), but solely due to strategic adjustments of eye movement behavior to the requirements of our perceptual task. However, there are several reasons for why we believe that the effects in most conditions of our experiments are due to oculomotor learning, and not a mere consequence of a strategic process. First, amplitude changes in most conditions of both experiments persisted to some degree in retention trials, where no candidate stimuli were shown, and where participants, thus, did not have to complete the perceptual task. After-effects like this are characteristic for saccade adaptation (43) and are expected to be absent if the observed amplitude changes were only driven by a strategic process (e.g., see Ref. 56). In other words, if amplitude changes were only due to participants strategically adjusting their eye movement behavior to ensure task-performance, they should disappear whenever the deployed strategy was no longer adaptive. Since participants were instructed that the presence or absence of candidate stimuli is constant in a block of trials, but can change between blocks, those after-effects are also not just a consequence of uncertainty about if candidate stimuli will be shown in a trial or not. Second, saccade adaptation affected the acceleration and deceleration phase of saccades differently. This effect is, again, characteristic for saccade adaptation, and this differential effect is absent when participants are instructed to target a certain on-screen location, i.e., when their eye movements are likely guided by only a strategic process (38). Finally, results of model fitting showed that the observed amplitude changes are not only driven by a strategic process, but also by a gradual learning process, which is typically used to model time courses of saccade adaptation (e.g., see Ref. 37).

Overall, results from our model fitting are in line with other findings from both, the literature on saccade adaptation (23–25, 45) as well as the literature on adaptation of reaching movements (18, 40, 46, 57). Especially for adaptation of reaching movements it is by now well established that two processes interact: an implicit process, which gradually corrects movement errors without awareness of participants, and an explicit process, which corresponds to behavior that participants strategically use to counteract imposed perturbations to movement accuracy. For example, one recent study reported that adaption of reaching movements is driven by an implicit and an explicit process, which utilize visual target errors to evaluate movement accuracy (40, see also Ref. 58). Depending on the testing environment, those two processes might become entangled, and compete to reduce one common source of error (40). Enhancing the response of one process, for example by providing instructions how to counteract imposed perturbations, can lead to a rapid depletion of movement error, i.e., the driving force for the other process. Thus, depending on the experimental conditions, one process might dominate over the other, and the contribution of an explicit process does not necessarily rule out an additional contribution of genuine motor learning, i.e., an implicit process.

An interaction between different learning processes was also observable across conditions of the small-target and large-target experiment and manifested in different adaptation time courses between experiments. This interaction was especially notable across conditions of the large-target experiment: for example, some participants in the discriminate-2nd condition of the large-target experiment showed amplitude changes toward the location of the task-relevant candidate stimulus, whereas other participants adapted toward the task-irrelevant candidate stimulus. Thus, different participants used different behavioral strategies to cope with the perceptual task in this condition, which highlights the relevance of strategic processes in our paradigm. Furthermore, across conditions of the large-target experiment, the strategic process compensated for most of the experienced movement error in adaptation trials, but significant after-effects were observable across most retention phases. This is reminiscent of an effect reported by Heins and Lappe (22), where gradual learning occurred throughout their saccade adaptation procedure, but this gradual learning was masked by the contribution of strategic oculomotor behavior, deployed to meet task-demands. Except for some notable examples (22–26, 45), studies on saccade adaptation to this day primarily focus on perturbing saccade accuracy by using the established double-step paradigm (7), where movement accuracy is perturbed without awareness of participants, to facilitate the contribution of a gradual process to oculomotor learning. Results from our two experiments, as well as results from the field of motor adaptation more broadly, point into a different direction, and highlight the relevance of studying saccade adaptation in more complex environments, where implicit and explicit processes can occur and interact.

One open question is how selection of error signals in dynamic environments is implemented on a neural level. It is well established that saccade adaptation, and motor adaptation more generally, depend on low-level brain structures such as the superior colliculus, where error signals are generated (e.g., see Ref. 59), and the cerebellum, where perturbations to movement accuracy are corrected (for reviews, see Refs. 4 and 11). However, some recent studies found evidence that the cerebellum is also involved in a variety of higher-level functions (for a review, see Ref. 60), such as reward processing (61, 62), temporal preparation in response to external cues (63, 64), shifting attention to enhance perceptual sensitivity (65), and maintenance of motor-independent representations of objects in the world (66). Thus, saccade adaptation in complex environments, where target selection is necessary to resolve conflicts between temporally asynchronous competing error signals, might be implemented by similar brain structures as saccade adaptation in simpler testing environments, where feedback about movement accuracy is immediate and unambiguous.

To conclude, we investigated how spatially accurate saccades are maintained in dynamic environments, where the availability of error signals varies dynamically over time. Our study revealed a complex influence of the visual environment, on both, which error signal the brain uses to evaluate movement accuracy, and how movement errors are corrected subsequently.

## DATA AVAILABILITY

Data and analysis code are available from the following doi: https://doi.org/10.5281/zenodo.7128739.

## SUPPLEMENTAL DATA

10.5281/zenodo.7128738 Supplemental Figs. S1 and S2 and Supplemental Tables S1 and S2: https://doi.org/10.5281/zenodo.7128738.

## GRANTS

A. C. Schütz and I. Wagner were funded by the Deutsche Forschungsgemeinschaft (DFG, German Research Foundation)—Project Number 222641018-SFB/TRR 135 TP B2 and the DFG International Research Training Group IRTG-1901-The Brain in Action. A. C. Schütz received funding from the European Research Council (ERC) under the European Union’s Horizon 2020 research and innovation programme (Grant Agreement No. 676786) and from “The Adaptive Mind,” funded by the Excellence Program of the Hessian Ministry of Higher Education, Science, Research and Art.

## DISCLOSURES

No conflicts of interest, financial or otherwise, are declared by the authors.

## AUTHOR CONTRIBUTIONS

I.W. and A.C.S. conceived and designed research; I.W. performed experiments; I.W. analyzed data; I.W. and A.C.S. interpreted results of experiments; I.W. prepared figures; I.W. drafted manuscript; I.W. and A.C.S. edited and revised manuscript; I.W. and A.C.S. approved final version of manuscript.
